# Royal Jelly as a Nutraceutical Natural Product with a Focus on Its Antibacterial Activity

**DOI:** 10.3390/pharmaceutics14061142

**Published:** 2022-05-27

**Authors:** Lilla Bagameri, Gabriela-Maria Baci, Daniel Severus Dezmirean

**Affiliations:** Faculty of Animal Science and Biotechnology, University of Agricultural Sciences and Veterinary Medicine Cluj-Napoca, 400372 Cluj-Napoca, Romania; ddezmirean@usamvcluj.ro

**Keywords:** royal jelly, natural product, antibacterial activity, antioxidant effect, anticancer activity, antibiotic resistance

## Abstract

Royal jelly (RJ) is one of the most valued natural products and is known for its health-promoting properties. Due to its therapeutic effects, it has been used in medicine since antiquity. Nowadays, several studies indicate that RJ acts as a powerful antimicrobial agent. Indeed, researchers shed light on its antioxidant and anticancer activity. RJ’s biological properties are related to its bioactive compounds, such as proteins, peptides, phenolic, and fatty acids. The aim of this review is to highlight recent findings on RJ’s main bioactive compounds correlated with its health-promoting properties. The available literature suggests that these bioactive compounds can be used as an alternative approach in order to enhance human health. Moreover, throughout this paper, we underline the prominent antibacterial effect of RJ against several target bacterial strains. In addition, we briefly discuss other therapeutic activities, such as antioxidative and anticancer effects, of this outstanding natural product.

## 1. Introduction

Antibacterial resistance poses a challenging threat to global public health. Due to the exponential growth of multidrug-resistant pathogens, there is an urgent need to explore alternative antibacterial agents. Royal jelly (RJ) is one of the most studied bee products, gaining popularity due to its health-promoting benefits. This natural product’s pharmacological properties have been widely investigated from animal models to human [1]. It is a white to yellowish viscous bee product with a sharply strong odor and taste [2], secreted by the mandibular and hypopharyngeal glands of worker honeybees [3]. It represents an exclusive form of nourishment for the queen honeybee during its lifetime and also it is fed to all larvae in the first three days of their maturation [2,4]. After this three-day period, nurse bees start to eat a mixture of honey and pollen, called worker jelly [2]. RJ is an acidic colloid, with a pH value ranging from 3.6 to 4.2 [5,6], and comprising 9–18% proteins (*w*/*w*) [7,8], 3–6% lipids (*w*/*w*) [5] and 0.8–3% (*w*/*w*) other compounds, such as free amino acids and fatty acids [6,9]. Due to the fact that it possesses various pharmacological properties, RJ is employed as a natural agent in medical fields, as well as in cosmetic and food industries [1]. Among these, antimicrobial [10,11], antioxidant [12], anti-inflammatory [3], antitumoral [13], antiviral [14], immunomodulatory [15], neuroprotective [13], antiaging [16] properties were reported. Data in the current literature state that RJ has the highest market value among bee products [1]. From a well-managed hive, about 500 g of RJ could be obtained during the summer season [17,18]. In this regard, the improvement of its production exhibits interest for many beekeepers. China is considered to be the largest RJ producer and exporter worldwide, with an estimated annual production exceeding 4000 tons of RJ, accounting for about 90% of the total amount harvested globally [1,9].

We aimed to summarize the progress of using RJ as a therapeutic product against a wide spectrum of bacterial pathogens which cause life-threatening infections. Furthermore, we review the correlation between RJ’s bioactive compounds and antibacterial activity. In this respect, special focus was applied to RJ’s microbial activity against *Pseudomonas aeruginosa*, *Escherichia coli*, *Staphylococcus aureus*, *Listeria monocytogenes*, and beyond. This review briefly summarizes research studies that highlight the antioxidative and anticancer activities of RJ, involving both animal and human trials. 

## 2. Insights into the Composition of RJ

**Figure 1:**
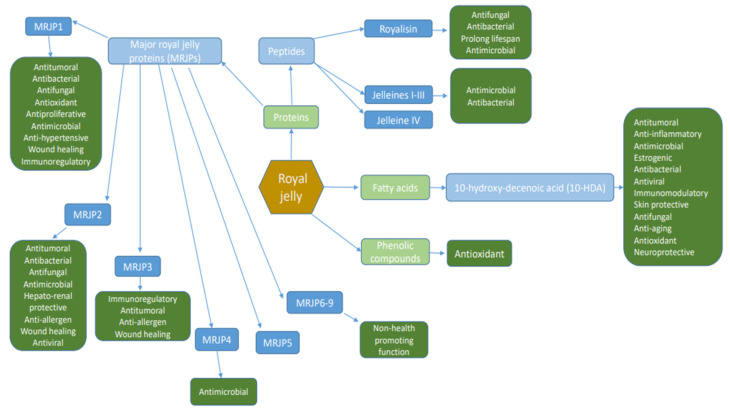
A schematic representation of RJ’s bioactive compounds and their functional activities [1,3,5,7,10,11,12,16,21,27,28,29,30,40].

Due to the heterogeneous nature and geographical origin of RJ, different values from different researchers have been obtained. Being a rich source of bioactive compounds (Figure 1), it is considered to be one of the most valued functional foods. 

### 2.1. Proteins

Proteins are key elements that represent the basis of RJ’s structure. These complex components constitute more than 50% of RJ’s dry matter [11]. Furthermore, more than 80% of protein content is represented by the group of major RJ proteins (MRJPs) [7]. According to Maghsoudlou et al. (2019) [19], nine MRJP proteins have been identified and ranged with respect to their molecular weight from 49 to 87 kDa. MRJPs are assumed to be responsible for the queen honeybee and larvae development by providing them a certain essential amino acid intake [13], including arginine, histidine, isoleucine, leucine, lysine, methionine, phenylalanine, threonine, tryptophan and valine [20].

MRJP1 is the major RJ glycoprotein and its architecture includes both monomeric (55 kDa) and oligomeric forms. The oligomer configuration (280–420 kDa), called apalbumin [5,11], exhibits a negatively charged surface, even at a neutral pH value. However, the monomeric form, specifically royalactin, displays lower heat resistance and shorter storage stability compared to apalbumin [21]. Besides its great role in honeybees, MRJP1 has a wide range of pharmaceutical effects on human health, such as wound healing and antibacterial [22], antifungal [1], hypocholesterolemic [23], antitumor [24] and immune-enhancement activities [25].

According to Lin et al. (2018) [26], MRJP1 plays an essential role in RJ quality determination, as well as in RJ freshness. This feature varies depending on storage conditions. Storage above 4 °C triggers the degradation of MRJP1 due to its trypsin action. The degradation process can be measured by the enzyme-linked immunosorbent assay (ELISA) [19]. In contrast to MRJP1, MRJP2 is present exclusively in monomeric form (50–59 kDa), representing a 435 residue-long glycoprotein. It has been proven that it is responsible for the antibacterial activity of RJ against *Paenibacillus larvae.* The antibacterial activity of MRJP2 has been linked to its peptide content, which mainly encompasses apidaecin and hymenoptaecin [27]. Additionally, MRJP2 exhibits antibacterial activity against B. subtilis and *E. coli* because of its high mannose carbohydrate content [28]. MRJP3 is 524 residue-long [19] polymorphic protein [29] with a 60–70 kDa molecular weight. This protein is recognized due to its nitrogen supply ability [19]. MRJP 1–5 are responsible for the essential amino acid intake, while MRJP2, MRJP3 and MRJP5 provide the nitrogen supply [30]. In contrast, it was reported that MRJP 6–9 do not have nutritional value [11,31]. Several studies indicate that RJ should be stored in a frozen state in order to prevent the decomposition of bioactive compounds [7,15].

#### Antimicrobial Peptides

The antimicrobial peptides (AMPs) are key players for various living organisms’ innate immune systems [32]. AMPs exhibit a wide spectrum of therapeutic activities, including antibacterial, antiviral [16], antitumoral [33], immunoregulatory and hepatoprotective activities [16]. These structures are cationic and amphiphilic molecules [34] and are identified in diverse species, such as animals, plants, bacteria, but also are found in several species of fungi. AMPs are cationic due to the presence of arginine (6.48 ± 0.04), lysine (15.93 ± 0.89) and histidine (2.91 ± 0.01) residues, which grant them the ability to interact with anionic phospholipids [35]. As cationic AMPs interact with the negatively charged cell membrane of various microorganisms, they result in an electrochemical change. This process leads to a destruction of bacteria integrity, thus to the death of bacterial cells [16].

Regarding AMPs, one of the most important players is the group of defensins, including cysteine-rich AMPs. Royalisin is a key member of the family of AMPs that is found in RJ. It is an amphipathic peptide, rich in amino acids, accounting for about 6.5% of RJ’s total protein content [36]. The primary structure of royalisin consists of 67 residues with three intramolecular disulfide linkages, having a molecular mass of 5.5 kDa and 51 amino acids, which provide it with high stability at low pH and high temperature [37].

Another class of AMPs that has great importance for RJ’s antimicrobial activity is the group of elleines. These peptides are considered to be the antimicrobial peptide family present in the C-terminal portion of MRJP1 [38]. Jelleines I, II, and III were found to have antimicrobial activities against yeast, Gram-positive and Gram-negative bacteria. On the other hand, jelleine IV does not show antimicrobial activity [5]. Even though they possess similar structure and functions, jelleines do not present any similarity with other antimicrobial peptides [11]. The content of these peptides can be determined by using numerous techniques such as the reverse-phase high-performance liquid chromatography (HPLC) method [39].

In order to determine the protein content, ISO recommends the Kjeldahl method. Even so, there are many more methods used in various studies, such as sodium dodecyl sulfate polyacrylamide gel electrophoresis and polyacrylamide gel electrophoresis, that are mainly employed in combination with mass spectrometry, as well as with gel-free proteomics [19]. Furthermore, Lowry and Bradford methods are also considered adequate analyses [13]. In order to determine the isolated proteins, several studies applied chromatographic methods, such as ion exchange chromatography, gel filtration chromatography and high-pressure liquid chromatography (HPLC). These methods are based on the adsorption, dispersion, ion exchange, affinity and molecular weight differences [20].

### 2.2. Lipids

When it comes to lipids content, these macromolecules represent 7–18% of RJ bioactive compounds [40] and 3–19% of RJ dry matter [41]. The lipid content encompasses more than 80% of fatty acids, phospholipids (0.4–0.8%), phenols (4–10%), steroids (3–4%) and waxes (5–6%). Although they can only be detected in a trace amount, sterols are also involved in RJ’s lipid fraction, composed mainly by 24-methylene cholesterol (24-MET) [1]. Medium chain fatty acids (MCFAs) contain 8–12 carbon atoms, predominantly hydroxyl and dicarboxylic forms [42,43,44]. Long-chain fatty acids are absorbed and transported to the liver less efficiently than MCFAs [45]. More than 80% percent of the total lipid content is represented by fatty acids, among which 10-hydroxy-2-decenoic (10-HDA) is the most popular [42,43]. Free fatty acids contain 8–12 carbon atoms, predominantly hydroxyl and dicarboxylic forms [44]. RJ also contains sebacic acid, 8-hydroxyoctanoic acid (8-HOC), 3,10-dihydroxydecane-dioic acid (3,10-HDecDA), 9-hydroxy-2-decenoic acid (9-HDA), 3-hydroxydecanoic acid (3-HHDA), 10-hydroxydecanoic acid (10-HDAA), 10-hydroxy-2-decenoic-acid (10-H2DA), and 2-decene-1,10-dioic acid (2-DecDA) [1,43]. Among fatty acids, 10-hydroxy-2-decenoic (10-HDA) is the most popular [42].

10-HDA is an unsaturated fatty acid that is found exclusively in RJ. It exhibits several pharmacological effects, such as antitumoral, immunomodulatory, and antibacterial [5]. The amount of 10-HDA in pure RJ varies being directly influenced by the harvesting period; thus, the highest mean value in RJ harvested in April and June is 1.84%; meanwhile, the lowest is 1.03% [46]. According to Fratini et al. (2016) [29], 10-HDA is the main criterion for determining RJ quality and freshness. Based on the standards of the International Organization for Standardization (ISO), the total amount of 10-HDA should be more than 1.4% for pure RJ in order to meet quality control parameters (ISO, 2016). About 3–6% of lipids have been identified as responsible for important biological activities related to the development strategies of the colony. Furthermore, lipid content analysis plays a key role in determining authenticity due to the fact that an adulterated RJ presents a lower amount of protein and lipid [29].

As for the ISO recommended methods for the 10-HDA content determination, HPLC-UV External represents the reference method and HPLC-UV is the internal standard as an alternative method [47].

### 2.3. Carbohydrates

As for the carbohydrates present in RJ, they represent about 30% of RJ’s dry matter, and play a major role in RJ’s authenticity [29]. Fructose and glucose account for 90% of the total sugar content in RJ, with an average concentration of 2.3–7.6% (mean 4.9%) as well as 2.9–8.1% (mean 5.5%). Huge differences have been observed in the content of sucrose, being identified in variable concentrations and ranging <0.1–3.6% (mean 0.6–1.87%) [11]. Even though constituents such as trehalose, maltose, erlose, melibiose, ribose [40], gentiobiose, isomaltose, raffinose, and melezitose are present in a small quantity, they play an essential role in determining bee products authenticity [48]. According to Kunugi and Mohammed-Ali (2019) [40], RJ sugar content highly depends on season, bee species, as well as on geographical and botanical origin. However, Nath et al. (2019) [49] did not find a remarkable difference in sugar content depending on the flower source. On the other hand, Popescu et al. (2009) [50] showed that sucrose content varies depending on collected samples. According to ISO, the sugar content is determined through liquid chromatographic analysis (the reference method), titration method and gas chromatographic analysis [51].

### 2.4. Phenolic Compounds

The leading group of plants’ secondary metabolites is phenolic compounds. These elements are in the spotlight due to their correlation with nourishment, but also with several therapeutic effects, such as antibacterial and antioxidative activities. Phenols are heterogeneous bioactive compounds and regarding their chemical structure they include one or more aromatic rings with a certain number of hydroxyl constituents. Depending on the number of phenol units, the phenolic compounds are divided in two main classes; thus, there are simple phenols that include a single phenolic constituent and polyphenols that comprise multiple phenolic elements [5,52,53,54,55,56]. The phenolic compounds possess different mechanisms of antimicrobial action. They act by inhibiting the synthesis of nucleic acids, decreasing the membrane fluidity, and suppressing microbial virulence factors. Furthermore, they were shown to work synergistically with antibiotics, thus increasing its efficacy and decreasing the necessary dose [57].

Polyphenols are considered ubiquitous compounds that possess antioxidant activities by stabilizing free radical scavengers. Moreover, they counteract the effects of free radicals due to their redox properties [14]. Due their complex molecular structure, they exhibit various antimicrobial effects, including inhibition of extracellular enzyme or permeabilization and destabilization of the plasma membrane. In addition, they can inhibit the growth of a variety of microorganisms, such as bacteria, protozoa, food-related pathogens, and fungi [57]. The bioavailability of polyphenols is highly impacted by the environmental conditions and the nourishment source. Furthermore, there are other elements that influence this parameter of polyphenols, for instance, the chemical composition, but also the interaction between these micronutrients and proteins and beyond [58]. According to numerous studies, the total polyphenol content can be determined by the Folin–Ciocalteu colorimetric method [59,60,61,62,63].

One of the most important groups of polyphenolic compounds found in bee products is the class of flavonoids. The total flavonoid content in RJ is 1.28 ± 0.09 rutin equivalent (RE) μg/mg; meanwhile, the proportion of phenolic compounds is 23.3 ± 0.92 gallic acid equivalent (GAE) μg/mg [40], but this amount may vary depending on the food source [3]. With respect to their structural complexity, they are subdivided into flavones (acacetin, apigenin, chrysin, and luteolin), flavanones (pinobaskin, pinocembrin, hesperidin, naringin, and naringenin), flavonols (fisetin, galangin, isorhamnetin, kaempferol, quercetin, and rutin), isoflavonoids (coumestrol, formononetin, and genistein) [1,42], anthocyanins, and chalcones [64].

### 2.5. Vitamins and Minerals

One of the greatest advantages of RJ as a natural product is its vitamin content. RJ is exceptionally rich in vitamins, especially in B-complex vitamins, among which, notably, pantothenic acid (vitamin B5) and niacin (vitamin B3) are present in the highest amount. Additionally, folic acid and ascorbic acid (vitamin C), vitamin D, E and A are present in trace amounts [9,11,65]. According to Fratini et al. (2016) [29], vitamin content can present changes depending on seasonal and floral variations of the pollen.

Minerals constitute about 4–8% of RJ’s dry matter [29]. RJ contains a wide range of minerals, such as potassium, phosphorus, sulfur, calcium, magnesium, iron, sodium, zinc, copper, chromium, cadmium, and palladium [11,66]. It is well known that potassium has important functions in the human body. One of the most important roles of potassium is the regulation of the fluid balance, but also it manages the heart’s electrical activity. Among these functions, this essential mineral also plays a key role in reducing blood pressure, thus preventing the risk of stroke [67,68]. According to Fratini et al. (2016) [29], the mineral content is influenced by the environmental factors and the production season, but also it depends on several bee’s biological characteristics and the feed source.

### 2.6. Water

As in other natural products, RJ possesses a high amount of water (more than 60%), which may be an assurance of a constant moisture content inside the hive. Water quantitative analysis plays a key role in the determination of RJ’s quality [69]. There are several factors that exhibit impact on water content, such as processing approaches, but also the season of production. However, storage conditions also have an influence. Vacuum drying oven (the reference method) [51], Karl Fischer titration, and lyophilization are the main recommended determination methods by the ISO [70]. Alternatively, infrared and refractometric methods were used by Maghsoudlou et al. (2019) [19] in order to determine RJ quality.

## 3. RJ as an Antimicrobial Agent

Antibiotic-resistant bacteria pose a serious threat to human health worldwide. Thus, finding and developing up-to-date antibacterial agents that counteract their spread are gaining popularity. Being a rich source of bioactive compounds, bee products, such as RJ, honey or propolis are considered to be a natural alternative to conventional antibiotics. RJ is known to be effective against various bacteria that cause life-threatening infections both in humans and animals [29].

Oral cavity bacterial infections are an increasing problem, with nearly 3.5 billion people affected worldwide. Periodontal diseases, including gingivitis and periodontitis, are considered to be the main causes of tooth loss [71]. Conventional medication, such as antibiotic treatment, exhibits side effects, among which toxicity and allergies are the most common. In addition, it is well known that a wide range of bacterial strains are resistant to antibiotics’ action. Keeping this in mind, nowadays, natural treatment alternatives, such as apitherapy, have gained high interest [72]. Khosla et al. (2020) [73] evaluated RJ’s antibacterial activity against periodontopathic bacteria by analyzing subgingival plaques. The authors divided the pathogens into aerobic (*Porphyromonas gingivalis*, *Prevotella intermedia*, *Tannerella forsythia*, *Treponema denticola*, *Fusobacterium nucleatum*) and anaerobic bacteria (*Aggregatibacter actinomycetemcomitans*). They aimed to evaluate and compare the antimicrobial properties of RJ and chlorhexidine against the mentioned bacteria. In this study, 15 patients with chronic periodontitis were enrolled, each of them receiving a dose of 0.6 g RJ. The results of their study concluded that RJ exhibits higher antibacterial activity against anaerobic bacteria than aerobic ones. In addition, they shed in light the fact that RJ has minimal side effects; thus, it can be considered a potential alternative to synthetic antimicrobials. Furthermore, Coutinho et al. (2018) [72] analyzed the antimicrobial effect of RJ against several bacterial strains, namely, *A. actinomycetemcomitans*, *P. gingivalis*, *Prevotella intermedia* and *F. nucleatum*. The authors reported that all the tested bacterial strains were sensitive to RJ. Two of the bacteria, specifically *P. gingivalis* and *P. intermedia*, were resistant to RJ at a concentration ranging 0.2–100 µg/mL, whereas *F. nucleatum* and *A. actinomycetemcomitans* were sensitive to RJ at 0.2–6.25 µg/mL values. It was stated that minimum inhibitory concentration (MIC) values ranged 0.2–12.5 µg/mL. In addition, minimum bactericidal concentration (MBC) values have been tested, resulting in a bactericidal effect that ranged 12.5–100 µg/mL. The authors related this bioactive effect to the 10-HDA, protein and peptide content, which plays a key role in RJ’s action mechanism. They attributed this bioactive effect to the protein content, which plays a key role in the defensive mechanism.

According to the European Food Safety Authority (EFSA), in 2020, there were reported 1876 cases of illness, 780 hospitalisations and 167 deaths in the EU [74]. Recently, Altunaș et al. (2020) [75] carried out a study on RJ as a potential anti-biofilm agent against *L. monocytogenes*, a common food pathogen. Biofilms pose a threat especially to alimentary industries, due to the fact that they are feasible contaminants of food and drinks. *L. monocytogenes* is a Gram-positive foodborne bacterium with a mortality rate of 20–30%. The authors evaluated for the first time the effects of RJ against *L. monocytogenes*. In the study, there were assayed different concentrations of RJ. The results show that RJ inhibited the formation of the biofilm at 0.33 mg/mL. Moreover, an increased concentration (from 0.33 mg/mL to 41.67 mg/mL) provides a higher protection against the biofilm formation.

Bilikova et al. (2015) [37] evaluated royalisin and royalisin-D antibacterial activity against both Gram-positive and Gram-negative bacteria. The authors assayed two bacterial strains, namely, *S. intermedius B* and *P. aeruginosa*. They reported that royalisin acts against bacteria survival by decreasing their cell surface hydrophobicity from 56–63% to 13.7–18.2%, while royalisin-D reduces it from 56–63% to 20.4–25.1%. The cell membrane permeability of the tested bacteria was evaluated by UV absorbance at 260 nm. Researchers concluded that both of the peptides possess antibacterial activity against a broad spectrum of Gram-positive bacteria. Furthermore, they registered weak activity against Gram-negative bacteria and fungi. Their result indicated that royalisin-D displays lower antimicrobial activity than royalisin, whose bioactive activity is associated with its intramolecular disulfide linkage.

Melliou and Chinou (2005) [76] studied the antibacterial activity of Greek RJ both against Gram-positive (*S. aureus*, *S. epidermidis*) and Gram-negative bacteria (*E. coli*, *Enterobacter cloacae*, *Klebsiella pneumoniae*, *P. aeruginosa*). The authors attributed RJ’s antibacterial activity to fatty acids, especially 10-HDA. As mentioned by Kunugi and Mohammed-Ali (2019) [40] as well, 10-HDA acts as a strong bactericidal, due to RJ’s low pH level. Sediva et al. (2018) [77] demonstrated that the inhibitory activity of 10-HDA increased at a low pH level, assayed on *Paenibacillus larvae*. Researchers stated that this compound can be used in order to protect bee larvae against several infections that can have a negative impact above the beehives.

According to many researchers, RJ possesses high antimicrobial properties against a broad spectrum of pathogens due to the existence of bioactive compounds, such as proteins, peptides and fatty acids [1,11,27,72,77].

### 3.1. RJ against P. aeruginosa

The genus Pseudomonas is one of the most studied and complex genera of the microbial taxonomy and involves numerous Gram-negative bacterial species. The P. spp. are known for playing key roles in various scientific areas, such as ecology or biotechnology. However, there are species that represent a threat for humans’ health [78]. One of the most analyzed opportunistic pathogens that belongs to the genus Pseudomonas is *P. aeruginosa*. It is exhaustively distributed, being detected not just in abiotic conditions, but also in biotic conditions. *P. aeruginosa* can be found and isolated from aquatic environments, medical devices to human tissues, plants [79,80], and beyond [81]. It is widely distributed due to its great adaptability, being able to live in various conditions, and requires minimal sources of nourishment [82]. Furthermore, *P. aeruginosa* is one of the most common infectious pathogens that causes nosocomial infections. When it comes to invasive infection in burns, *P. aeruginosa* is the most common pathogen. It is necessary to inhibit its growth in order to prevent life-threatening burn infections [83,84]. Several studies showed that this microorganism has a great capacity to develop resistance against numerous antibiotics [85,86,87,88,89,90] through various mechanisms [86,91,92,93]. This pathogen represents the source of a broad spectrum of chronic infections. The most susceptible individuals to *P. aeruginosa* are the immunocompromised patients; therefore, its high pathogeny is associated with comorbidities such as cystic fibrosis, cancer or burns [94].

Keeping in mind the fact that *P. aeruginosa* is one of the most common pathogens that leads to nosocomial infections in immunosuppressed patients and its high resistance to antibiotics, it is necessary to develop new effective antibiotics. When it comes to invasive infection in burns, *P. aeruginosa* is the most common pathogen. It is necessary to inhibit its growth in order to prevent life-threatening burn infections [83,84]. However, the process of extending the range of antibiotics is limited. Therefore, natural products are in the spotlight due to their therapeutics activities [95]. One of the most studied natural remedies against *P. aeruginosa* is RJ; thus, numerous studies have demonstrated its antibacterial activity [96].

Amly et al. (2021) [97] investigated the antibacterial activity of RJ against *P. aeruginosa*. The authors highlighted the potential of RJ to combat the resistance of this pathogen to antibiotics. In this study, the honeybee product was used against *P. aeruginosa* ATCC^®^ 10145™, but also in cases with clinical isolates. Their data showed that by inoculating *P*. *aeruginosa* suspension in a tube that contained LB broth culture medium and RJ with a concentration of 25%, microorganism growth was restrained, this being the minimum inhibitory concentration (MIC). Furthermore, they explored the effect of RJ’s subinhibitory concentration on *P. aeruginosa*. Their results revealed that a subinhibitory concentration exhibits effect on pyocyanin. This represents a secondary metabolism product—specifically, a cytotoxic natural pigment. Pyocyanin is used in order to determine the pathogenicity of this microorganism [97]. However, pyocyanin has garnered great interest from numerous industries, medicine and agriculture as a colorant agent [98]. By analyzing the effect of RJ on pyocyanin production, they concluded that three concentrations of the natural product, namely, 3.125%, 6.25% and 12.5%, led to pyocyanin production expansion of the ATCC^®^ 10145™ strain. However, pyocyanin production in the clinical isolates was significantly stimulated by using RJ concentrations of 6.25% and 12.5%. Their results are of great interest not only for the purpose of overcoming the antibiotic resistance crisis, but also for choosing the relevant concentration of RJ in order to increase or decrease the pyocyanin concentration.

It has been previously shown that the antimicrobial peptide named jelleine-I exhibits great potential as an antifungal therapeutic agent [99]. However, the same research group aimed to investigate the antimicrobial activity of jelleine-I against several bacterial agents. Among the chosen pathogens, the antibacterial activity of jellein-I was exploited against *P. aeruginosa*. The authors demonstrated that jelleine-I has an inhibitory effect against this certain pathogen, and the development of antimicrobial agents that involve jelleine-I is feasible [100]. Moreover, Zhou et al. (2021) [101] developed several jelleine-I analogs that exhibit intensified antimicrobial effects against *P. aeruginosa*. By investigating the activity of the developed analogs, the authors concluded that one certain analog, that expresses in its sequence additional arginine and leucine, displays improved antibacterial effect against *P. aeruginosa*.

Taken together, these studies highlight the potential of RJ to inhibit the growth of *P. aeruginosa*. However, not only does RJ suppress bacterial development, but it has been shown that this outstanding natural product exhibits great ability in preventing and inhibiting the adhesion property of *P. aeruginosa*. Its adhesion property plays a key role in pathogenesis, being a significant factor in the inhibition process [102].

### 3.2. RJ against E. coli

*E. coli* is a Gram-negative anaerobic bacterium that has extensive distribution. When it comes to mammal intestinal microbiota, *E. coli*. is the main facultative anaerobic bacterium, being an opportunistic pathogen [103,104,105]. However, there are several pathogenic strains that can lead to both intestinal and extraintestinal pathologies, and affect not only the immunocompromised patients, but the healthy individuals [104]. Furthermore, *E. coli* has drawn extraordinary levels of interest from the scientific community due to its great roles; specifically, it represents a powerful tool for the production of important pharmaceutical agents [106], but it is also used as an indicator for monitoring the antimicrobial resistance [107]. Numerous studies certified that *E. coli* developed antibiotic resistance against a broad spectrum of antimicrobial agents [108], but also represents the most common infectious microorganism responsible for neonatal infections [109]. To tackle resistance against certain antibiotics, numerous studies that use natural products have been performed in order to combat this global crisis [110]. In terms of this, RJ is one of the most promising natural products because it exhibits great levels of antimicrobial activity. For instance, Al-Abbadi et al. (2019) [111] explored the antiseptic activity of two types of RJ, namely, Chinese (CRJ) and Jordanian RJ. The researchers investigated this certain effect of the natural product against several bacterial species, including two *E. coli* strains, *E. coli* ATCC (0157) and *E. coli* ATCC (29522), respectively. The antimicrobial effect was analyzed by employing the well diffusion method. By using this method, the authors confirmed the antibacterial activity of RJ [106].

Hasan et al. (2020) [6] investigated the antibacterial activity of three antimicrobial agents, namely, doxycycline, gentamicin, and a combination of the two antibiotics, against *E. coli*. The authors compared the effect of these selective antibiotics with the antibacterial effect of RJ against the Gram-negative bacteria. Their results show that *E. coli* is not susceptible to the action of gentamicin, but it was also not inhibited by the combination of gentamicin and doxycycline. However, when using doxycycline against the opportunistic pathogen, significant antimicrobial activity was observed. In addition, RJ exhibited noteworthy antimicrobial activity as doxycycline against *E. coli*, highlighting the great potential as an antimicrobial natural product.

It is well known that MRJP 2 exhibits prominent antimicrobial effects. However, Park et al. (2019) [112] investigated the antimicrobial activity of the rest of MRJPs against *E. coli*. The authors developed recombinant MRJPs 1–7 by using a baculovirus-based expression system in insect cells. The results of this study revealed that MRJPs 2–5 and 7 display consistent antibacterial activity; thus, these proteins have a significant contribution to RJ’s inhibitory effect on bacterial growth. In another study, a research group used *E. coli* in order to overexpress the gene that encodes the MRJP 4. The authors successfully sub-cloned the target gene into the bacterial cells [113].

Another piece of research focused on exploring the antimicrobial activity of a combination between RJ and starch. This activity was investigated against both *E. coli* and *S. aureus*. This combination was developed due the fact that, in low-income countries, RJ is not affordable. The source of the first opportunistic pathogen was an individual that was aching from diarrhea. First, they investigated and confirmed the antimicrobial activity of RJ against both pathogens. Secondly, the authors added starch to the media containing RJ, and observed that by adding the second compound, the MIC dropped significantly; however, when the RJ was not added in the medium, the starch did not exhibit antimicrobial activity against certain bacterial strains [114].

### 3.3. RJ against S. aureus

One of the most common opportunistic Gram-positive bacteria is *S. aureus*. The nose represents the main colonization site of this specific bacteria. However, two other sites are frequently colonized by *S. aureus*, namely, the perineum and the throat. Even if pathogenic potential due to colonization exists, there are cases when infections occur due to contamination of medical devices, such as catheters [115,116]. This bacterium is mainly responsible for skin infections; however, it can lead to acute necrotizing pneumonia and infective endocarditis [117]. At the time of *S. aureus*’s discovery, in 1884, the mortality rate produced by its infection was near 82%. Later, in the 1940s, even if most of the strains were susceptible to the action of penicillin, 25% of the *S. aureus* strains that caused nosocomial infections were resistant to this certain antibiotic [117]. However, a semisynthetic-derived penicillin, specifically methicillin, was developed and the infection of *S. aureus* was controlled. Even so, after two years, the isolation of methicillin-resistant *S. aureus* strains was reported [118]. There is an urgent need to develop novel antibacterial drugs but, keeping in mind the ability of *S. aureus* to develop resistance to antibiotics, alternative strategies that leverage natural products such as RJ, have been in the spotlight [119].

Being a critical problem worldwide, the negative impact of methicillin-resistant *S. aureus* has accelerated progress in developing new therapeutic agents. El-Gayar et al. (2016) [120] explored the potential of using RJ and garlic to obstruct the growth of *S. aureus* strains that are not susceptible to methicillin. By using an in vitro and in vivo strategy, this specific research investigated the effect of RJ and garlic in a biofilm formation, but the authors also studied how adhesion and invasion were influenced. The data of this study revealed that even if the garlic extract exhibited great impact on biofilm formation, it did not inhibit bacterial adhesion. However, RJ successfully inhibited both biofilm development and microorganism adhesion. The outcome of the in vivo evaluation showed that garlic manifests antiseptic activity and RJ has an extraordinary prospective as an antibacterial agent by completely eradicating the *S. aureus* strains that exhibited resistance to methicillin. Furthermore, not only were the antibacterial effects of RJ highlighted, but also its considerable ability to promote the process of wound healing [120].

In a novel study, Hassan et al. (2022) [121] explored and provided detailed insights on the potential of RJ as a dietary additive in fermented milk. The authors supplemented milk with three concentrations of RJ, 0.5%, 1% and 1.5%, respectively. This research has demonstrated that RJ as a milk additive has numerous advantages, such as diminishing time of fermentation and increasing viscosity. More significantly, the authors evaluated the impact of RJ on various biological activities, including the antibacterial effect. In order to enhance the antibacterial, antioxidative and anticancer activities, the results showed that the optimal concentration of the additive compound was 1.5%. Preceding the addition of RJ in fermented milk, numerous bacterial strains, including *S. aureus*, were used in order to determine the antimicrobial impact. The fermented milk showed no antimicrobial activity against *S. aureus*. The growth of this opportunistic pathogen was inhibited by supplementing milk with 1% and 1.5% RJ [121]. Within the context of the antibiotic-resistant bacterial strains, Dinkov et al. (2016) [122] investigated the antimicrobial effect of two bee products, namely, rape honey and RJ against *S. aureus* strains that are methicillin-resistant. Moreover, the authors also investigated the antibacterial effect of their mixtures. This study confirmed the antiseptic activity of the two bee products, but also of their combination.

It is commonly assumed that RJ has great antimicrobial activity; recent research determined the impact of this bee product as an immunomodulating agent. The study was conducted by using the nematode *Caenorhabditis elegans* as the model organism and *S. aureus* as an infection model. Their results revealed that if RJ supplementation occurs at the egg-hatching stage, it has great potential to protect the host organism against the bacterial pathogenicity. Additionally, it has been demonstrated that the modulation of the innate immunity materialized due to RJ’s impact on three signaling pathways: IIS/DAF-16, p38 MAPK, and Wnt, respectively. The bee product was also delivered to aged *C. elegans* individuals exposed to several bacterial strains, including *S. aureus*, in order to investigate RJ’s potential on prolonging survival time. Indeed, the study disclosed that RJ contributes to a delay of the immunosenescence phenomenon. These findings suggest that RJ has great potential as an immunomodulatory supplement; thus, it plays a key role in the medical area and beyond [123]. In Table 1, a wide range of bacterial and fungal strains are listed, against which RJ or certain RJ constituents possess antibacterial activity.

## 4. Other Therapeutic Effects of RJ

The current literature suggests that the therapeutic effects of RJ are not limited to antibacterial activity, but it also possesses great anticancer and antioxidative activities. Furthermore, there are numerous studies that highlight RJ’s role in longevity and fertility.

It is well documented that cancer is one of the leading causes of death worldwide. The main therapies for cancer are chemotherapy and radiotherapy; however, these treatments exhibit adverse events. Keeping this in mind, there is major need to develop new treatment strategies that do not involve secondary effects [130]. There have been numerous in vitro studies aiming to investigate the anticancer effect of RJ. For instance, Ayna et al. (2021) [131] analyzed the effect of RJ on HT-29 colon cancer cell lines. The authors explored its chemopreventive impact, but also the antiproliferative and antioxidative activities. As for the cell proliferation assay, their results showed that the viability of the target cancer cells was affected by RJ. Furthermore, they showed that antiproliferative activity is concertation dependent. In Table 2, the main studies that demonstrated the anticancer activity and protective effect of RJ against numerous types of cancers are briefly described.

According to Mofid et al. (2016) [142], more than 50% of patients with malignancies who are undergoing radiotherapy, hormone therapy, or chemotherapy experience cancer-related fatigue (CRF). CRF is a common symptom which has a negative impact on life quality; thus, its detection in early stages is of great importance. It is often related to other symptoms such as anemia, pain, sleep disturbances and distress. The authors aimed to evaluate the effectiveness of RJ and processed honey on the symptomatology. The researcher group administered 5 mL of RJ and processed honey twice daily to 26 patients, and 5 mL of pure honey under the same scheme. The patients were monitored during a one-month period. The results of this clinical trial suggest that supplementation of RJ combined with honey can alleviate CRF.

A randomized, double-blinded and placebo-controlled clinical trial conducted by Araki et al. (2018) [143] aimed to assess RJ’s protective effect against tyrosine kinase inhibitor (TKI)-induced toxicity in patients with renal cell carcinoma (RCC). RCC is considered one of the most common forms of cancer in the urology department. Although molecularly targeted therapy using TKIs is the first line recommended in RCC, it has several adverse effects. In this regard, RJ as a natural alternative therapy is of great importance. In this study, 33 patients with advanced RCC were enrolled, among which, 16 were in RJ-supplemented group and 17 subjects were in a placebo group. The RJ group received 800 mg RJ capsules three times/day during a three-month period, whereas the placebo group received starch capsules under the same scheme. The findings suggest that RJ had a protective effect against TKI-induced fatigue and anorexia.

Balkanska (2018) [144] investigated RJ by finding correlations between its bioactive chemical compounds and antioxidant activity. In order to evaluate the antioxidant capacity, the authors explored two methods, specifically, ferric-reducing antioxidant power (FRAP), and 1,1-diphenyl-2-picrylhydrazyl (DPPH). Researchers discovered that RJ’s antioxidant activity is resulted from its 10-HDA and polyphenol content. HPLC and Folin–Ciocalteu methods were used in order to determine these contents. Their confirmed strong correlations between the FRAP method and polyphenol content, as well as between the DPPH method and 10-HDA content.

Protein hydrolysates are commonly used as health-promoting and cosmetic agents. Their physiological activities strongly correlate with their enzyme specificity. Chiang et al. (2021) [145] carried out a study on RJ protein (RJP) hydrolysates bioactive effects, with a special focus on the inhibition of DNA and LDL oxidative damage. In their study, RJP were hydrolyzed by two enzymes, namely, alcalase and flavourzyme. The authors reported that both RJP and RJP hydrolysates possess a high number of bioactive compounds, such as amino acids, 10-HDA, phenolic and flavonoid acids, which give them antioxidant activities. They concluded that these bioactive compounds have a protective effect against DNA oxidative damage. Furthermore, they highlighted the inhibitory effects on low-density lipoprotein (LDL) oxidation.

Polycystic ovary syndrome (PCOS) is an endocrine disorder with a prevalence ranging 15–20% among the reproductive-aged women [146]. It is characterized by clinical features, such as hyperandrogenism and ovulatory dysfunction. Moreover, it is assumed to cause infertility [147]. PCOS exhibits a negative impact on life quality due to its physiological alterations, including anxiety and depression [148]. Hamid et al. (2020) [149] investigated RJ’s effects on PCOS animal models’ hormonal profile, correlated with its bioactive compounds. In their experimental study, three doses of RJ were used, specifically, 100 mg/kg, 200 mg/kg, and 400 mg/kg. After a four-week period of RJ supplementation, the authors revealed that the 200 mg/kg was the most adequate dose. Estrus cycle regularity, ovarian histology, and function, improved reproductive hormone levels (LH, T, FSH, E2), as well as ovarian oxidant-antioxidant status (MDA, TAC, GPx) are some of the beneficial effects attributed to RJs phytochemical and bioactive compounds. Furthermore, researchers speculate that these ovarian histological changes are attributed mainly to the royalactin action, which is assumed to play a key role in the stimulation of the queen honeybee’s reproductive organ development. Researchers reported the antioxidant activity of RJ by highlighting its anti-androgenic effect. They report the requirement for further studies in order to identify certain action mechanisms.

Caixeta et al. (2018) [150] carried out a study assessing natural products with hepatoprotective activity. Liver tissues are vulnerable to acute and chronic stress; thus, the authors aimed to improve the organism’s capacity to adapt to different stress conditions. Wistar rats were randomly allocated into no-stress, no-stress RJ-supplemented, stress and stress-supplemented groups, and during a fourteen-day period, 200 mg/kg of RJ was administered to them. Researchers observed that RJ supplementation triggered corticosterone level decrease and glycemia control. The results show that RJ exhibits a hepatoprotective effect against oxidative-stress-induced damage. Moreover, RJ significantly increased the antioxidant capacity of restraint and cold-stressed rats. The authors state that RJ’s antioxidant effect may be attributed to phenolic compounds, peptides, fatty acids, and vitamins.

## 5. Conclusions

The use of RJ for nutraceutical, cosmetical, and medical purposes has long been exploited worldwide. Its bioactive compounds, such as proteins, peptides, phenolics and fatty acids, play a significant role in maintaining human health. In this review, the antimicrobial properties of this bee product have been discussed. With bacteria resistance against antibiotics being a global concern, the need for natural alternatives triggered advances in RJ’s research. Based on the evidence in the literature, RJ could fight a wide spectrum of pathogenic bacterial strains. This substance has demonstrated its efficiency against *P. aeruginosa*, *E. coli*, *S. aureus*, *L. monocytogenes*, *P. larvae*, and beyond. Furthermore, studies revealed that it exhibits great antioxidant and antitumoral activity, as well as other pharmaceutical properties, such as immunomodulatory, estrogenic, anti-inflammatory, hepatoprotective, and neuroprotective effects. As RJ’s mechanisms of action are yet to be fully understood, it continues to trigger interest in further research. Overall, RJ has been proved to be a promising natural agent which can be used as an alternative therapy against a variety of life-threatening health conditions.

## Figures and Tables

**Table 1:** RJ antimicrobial and antifungal activities against target microorganisms.

RJ/RJ Constituent	Targeted Microorganisms	Reference
MRJP2	*P. aeruginosa* *P. larvae* *Ascosphaera apis* *Candida albicans*	[112]
MRJP4	*P. aeruginosa* *P. larvae* *A. apis* *C. albicans*	[30]
RJ water extract MRJP1	*Melissococcus plutonius* *Enterococcus faecalis* *Paenibacillus alvei* *Brevibacillus laterosporus* *Bacillus pumilus* *E. coli* *P. fluorescens*	[22]
N-glycosylated MRJP2	*P. larvae*	[124]
10-HDA	*P. larvae*	[77]
10-HDA	*S. aureus* *Streptococcus alactolyticus* *Staphylococcus intermedius B* *Staphylococcus xylosus* *Salmonella cholearasuis* *Vibro parahaemolyticus* *E. coli*	[125]
RJ solution	*Bacteroides fragilis* *Bacteroides thetaiotaomicron*	[126]
10-HDA	*S. aureus*	[127]
Fresh RJ	Human gut microbiome	[128]
RJ extract	*Aspergillus parasiticus*	[129]

**Table 2:** The anticancer activity and protective effect of RJ against cancer treatments on different model organisms.

Activity	Cell Line/Model Organism	Type of Cancer	Reference
Antitumor growth	Mice	Breast cancer	[132]
Protective	Rats	Neck and head cancers	[133]
Protective	Rats	Prostatic cancer	[134]
Antioxidative	Rats	Breast cancer	[135]
Protective	Rats	Numerous types of cancer	[136]
Cytotoxic	PC3 cell line	Prostate cancer	[137]
Antiproliferative	SMMC-7721 cell line	Hepatocellular carcinoma	[138]
Antiproliferative	MCF-7 cell line	Breast cancer	[139]
Protective	Rat	Numerous types of cancer	[140]
Cytotoxic	HCT-116 and SW-480 cells	Colorectal cancer	[141]

## Data Availability

Not applicable.
